# Supplementary material for: MetaRibo-Seq measures translation in microbiomes
Source: Nat Commun. 2020 Jun 29;11:3268. doi: 10.1038/s41467-020-17081-z (PMC7324362; doi:10.1038/s41467-020-17081-z)
Supplement: Supplementary file 10 — Supplementary Data 7 [file 41467_2020_17081_MOESM10_ESM.zip › File2/Confidence_High_Taxonomy/421212_out.krona.html]

Javascript must be enabled to view this page.

members
magnitude
magnitudeUnassigned
count
unassigned
taxon
rank

421212\_out

30

30
superkingdom
2

30
1239
phylum

27
class
186801

27
186802
order

10
1898207

SRS012849\_contig\_number\_19571SRS013940\_contig\_number\_15172SRS075078\_contig\_number\_463SRS077194\_contig\_number\_contig-100\_105.145041SRS098717\_contig\_number\_19485SRS1041091\_contig\_number\_16029SRS1041136\_contig\_number\_10937SRS104636\_contig\_number\_6419SRS1054691\_contig\_number\_19747SRS893373\_contig\_number\_11948
species

1950935

SRS013098\_contig\_number\_23425SRS013965\_contig\_number\_26707SRS014855\_contig\_number\_6428SRS024663\_contig\_number\_15201SRS042628\_contig\_number\_15142SRS049896\_contig\_number\_11752SRS053356\_contig\_number\_33458SRS077392\_contig\_number\_12756SRS078419\_contig\_number\_11179SRS097889\_contig\_number\_11014SRS101433\_contig\_number\_contig-100\_145.95180SRS143466\_contig\_number\_26111SRS144714\_contig\_number\_27085SRS148721\_contig\_number\_32538SRS971275\_contig\_number\_8372
species
15

2
family
541000

1898205

SRS042966\_contig\_number\_16488
species
1

genus
216851
1

1
853

SRS020328\_contig\_number\_7438
species

3

SRS014613\_contig\_number\_19755SRS140492\_contig\_number\_24475SRS142890\_contig\_number\_21203
1262999
species
